# Latent Autoimmune Diabetes in Adults in the United Arab Emirates: Clinical Features and Factors Related to Insulin-Requirement

**DOI:** 10.1371/journal.pone.0131837

**Published:** 2015-08-07

**Authors:** Ernesto Maddaloni, Nader Lessan, Alia Al Tikriti, Raffaella Buzzetti, Paolo Pozzilli, Maha T. Barakat

**Affiliations:** 1 Department of Endocrinology and Diabetes, University Campus Bio-Medico, Rome, Italy; 2 Imperial College London Diabetes Centre, Abu Dhabi, United Arab Emirates; 3 Department of Experimental Medicine, “Sapienza” University of Rome, Rome, Italy; 4 Centre for Diabetes, The Blizard Building, Barts and The London School of Medicine, Queen Mary, University of London, London, United Kingdom; Weill Cornell Medical College in Qatar, QATAR

## Abstract

**Aims:**

To describe and to characterize clinical features of latent autoimmune diabetes in adults (LADA) compared to type 1 and type 2 diabetes in the UAE.

**Methods:**

In this cross-sectional study a dataset including 18,101 subjects with adult-onset (>30 years) diabetes was accessed. 17,072 subjects fulfilled the inclusion/exclusion criteria. Data about anthropometrics, demographics, autoantibodies to Glutamic Acid Decarboxylase (GADA) and to Islet Antigen 2 (anti-IA2), HbA1c, cholesterol and blood pressure were extracted. LADA was diagnosed according to GADA and/or anti-IA2 positivity and time to insulin therapy.

**Results:**

437 (2.6%) patients were identified as LADA and 34 (0.2%) as classical type 1 diabetes in adults. Mean age at diagnosis, BMI, waist circumference, systolic blood pressure and HbA1c significantly differed between, LADA, type 2 and type 1 diabetes, LADA showing halfway features between type 2 and type 1 diabetes. A decreasing trend for age at diagnosis and waist circumference was found among LADA subjects when subdivided by positivity for anti-IA2, GADA or for both antibodies (p=0.013 and p=0.011 for trend, respectively). There was a gradual downward trend in autoantibody titre in LADA subjects requiring insulin within the first year from diagnosis to subjects not requiring insulin after 10 years of follow-up (p<0.001).

**Conclusions:**

This is the first study describing the clinical features of LADA in the UAE, which appear to be different from both type 1 and type 2 diabetes. Furthermore, we showed that the clinical phenotype of LADA is dependent on different patterns of antibody positivity, influencing the time to insulin requirement.

## Introduction

Prevalence of diabetes is continuously increasing worldwide. In particular Middle East and North Africa region experienced a marked increase in the prevalence of diabetes. This has been quite dramatic among the affluent Gulf States. Specifically, the United Arab Emirates (UAE) has one of the highest prevalence rates of diabetes and pre-diabetes worldwide with official figures of 19.0% and 16.6% respectively in 2013 [1].

Even though type 2 diabetes is the most common form of adult-onset diabetes, autoimmune diabetes can also be diagnosed in the adulthood [2]. While newly diagnosed classical type 1 diabetes is relatively rare among adults, latent autoimmune diabetes in adults (LADA) accounts for up to 12% of all cases of diabetes in the adult population [3]. As a form of autoimmune diabetes, LADA is characterized by islet inflammation, suggested by the presence of one or more islet-specific autoantibodies, leading to the progressive loss of β-cells [4–6]. Differently from type 1 diabetes, LADA shows a slower progression towards β-cell failure and insulin requirement and higher levels of insulin resistance [7]. Thus, subjects affected by LADA are often misdiagnosed and wrongly treated as affected by type 2 diabetes [8].

Autoimmune diabetes has been described in Caucasian and Asian adults [9–12]. As genetic background, lifestyle, food habits and diabetes prevalence differ between various populations, so do the epidemiology and features of LADA among different peoples [13]. No data about prevalence and clinical features of LADA are available for Middle East and Gulf countries. The description of prevalence and features of adult-onset autoimmune diabetes in UAE could help in identifying more suitable prevention and treatment approaches and could also contribute to the pathophysiological knowledge of diabetes in this high-prevalence area.

In this study we aim to define the presence of adult-onset autoimmune diabetes in the UAE and to characterize clinical features of patients affected by LADA compared to type 1 diabetes and type 2 diabetes.

## Subjects and Methods

This single center study was conducted at the Imperial College London Diabetes (ICLDC). More than 50% of patients registered at the ICLDC are from Abu Dhabi, 26% from Al Ain and the remaining from other UAE cities including Bani Yas, Ras Al Khaimah, Sharjah, Al Shamkha and Dubai (S1 Table).

All patients attending the ICLDC are assessed by trained nurses and parameters, including the following anthropometric information, are obtained and recorded in the ICLDC database. Weight is recorded to the nearest 0.1 kg; height is measured with a stadiometer and recorded to nearest 0.5 cm; body mass index (BMI) as the ratio between weight in kg and the squared height in meters; waist circumference is measured using a metric non-stretching measuring tape, midway between the inferior margin of the lowest rib and the iliac crest in the horizontal plane at the end of normal expiration and recorded at the nearest 1.0 cm.

ICLDC patient database (accessed October, 2013) includes all patients attending the centre (n = 92,900), including those with diabetes mellitus (n = 34,886) and was accessed to identify patients with LADA.

### Identification of patients with LADA

18,101 subjects were identified as affected by adult-onset (>30 years) diabetes. Of these, we included in the study only those subjects with all the following information available at the time of the analysis (October 2013): measurement of autoantibodies to Glutamic Acid Decarboxylase (GADA) and to Islet Antigen 2 (anti-IA2); date of diabetes diagnosis; HbA1c, total cholesterol and blood pressure levels; time to insulin therapy; demographics and anthropometrics (date of birth, gender, height, weight, waist circumference). Subjects with age at diagnosis >70 years or with insufficient dataset were excluded from the study.

To determine factors influencing the initiation of insulin therapy in LADA subjects a sub-analysis was also conducted on subjects affected by LADA who started insulin before the date of data extraction or with disease duration longer than 10 years. Time to insulin therapy was calculated as the time between the date of diagnosis and the date of the first insulin treatment, as recorded in the database (need for insulin therapy was based on standard guidelines [14]).

Type 2 diabetes was diagnosed as non-autoimmune diabetes according to standard criteria [14]. Cases of LADA were defined as diabetic subjects with diabetes-associated autoantibodies who did not develop ketoacidosis or did not require insulin for at least 6 months after diagnosis [15]. Cases of type 1 diabetes were defined as case subjects with diabetes and diabetes-associated autoantibodies who did not fulfil criteria for LADA diagnosis (i.e. developed ketoacidosis or required insulin treatment within 6 months after diagnosis).

GADA and anti-IA2 were measured at the ICLDC Pathology Laboratory using enzyme-linked immunosorbent assays (Euroimmun AG, Luebeck, Germany), with the optimal cut-off value set at 10U/ml for both autoantibodies. Both assays have demonstrated good performance when tested in the Diabetes Antibody Standardization Program (DASP) Workshops 2005 (GADA: sensitivity of 92% and specificity of 98%; anti-IA2: sensitivity of 66%, specificity of 99%).

### Statistical analysis

Data are shown as mean ± standard deviation (SD) or standard error (SE) for continuous variables and as proportions for categorical variables. Student t-test and ANOVA test have been used to compare continuous variables between groups and Chi-square test to compare categorical variables. The non parametric Kruskal-Wallis test and the Fisher exact test have been used when appropriate. A p-value of less than 0.05 was considered statistically significant at 80% power. SPSS 21.0 for Windows was used to compute the analysis.

### Ethical Considerations

The study was conducted according to the principles expressed in the Declaration of Helsinki. All ICLDC patients signed a consent form allowing their anonymized information to be used for data analysis. Patient records were anonymized and de-identified prior to analysis.

## Results

One thousand and twenty-nine subjects with adult-onset diabetes were excluded from the study because of an incomplete dataset or age at diagnosis >70 years. A complete dataset was available for the remaining 17,072 subjects and their data were extracted and analysed (55.7% females; 98.6% arab; mean age at diagnosis 46.8 ± 9.6 years; mean BMI 31.3 ± 6.2 kg/m^2^; mean waist circumference 100.7 ± 12.6 cm; mean HbA1c at diagnosis 8.5 ± 2.1) (S1 File).

The prevalence of autoimmune diabetes was 2.8%. More specifically, 437 (2.6%) patients were identified as LADA and 34 (0.2%) as classical type 1 diabetes in adults. The highest proportion of diabetes diagnosis was made in subjects aged 40–49 years (proportions by age at diagnosis: 27.1% 30–39 years, 35.4% 40–49 years, 26.6% 50–59 years and 10.9% 60–69 years). Differently, the highest proportion of LADA diagnosis was in the third as opposed to fourth, fifth or sixth decade of life; LADA and type 1 diabetes were respectively diagnosed in 3.2% and 0.4% of all subjects diagnosed when they were in their thirties. The proportion of subjects affected by LADA and type 1 diabetes progressively decreased through forties (2.5% and 0.1% respectively), fifties (2.2% and 0.1%) and sixties (1.9% and 0.1%).

### Comparative features between type 2 diabetes, LADA and type 1 diabetes

Table 1 shows clinical and metabolic features of the three study groups. A significant decreasing trend from type 2 diabetes (highest value) to LADA (midway) and to type 1 diabetes (lowest) was found for mean age at diagnosis, BMI and systolic blood pressure, while an increasing trend was found for HbA1c. The proportion of subjects with family history of diabetes was higher in subjects with type 1 diabetes than in those with type 2 diabetes, while no significant differences were found between LADA and type 2 diabetes. Subjects affected by LADA differed from subjects with type 2 diabetes for age at diagnosis, BMI, HbA1c and systolic blood pressure, while they differed from subjects with type 1 diabetes for age at diagnosis, BMI, waist circumference, HbA1c, systolic and diastolic blood pressure. As expected, subjects with type 1 diabetes showed higher mean GADA and anti-IA2 titre levels (S2 Table).

**Table 1:** Population features by study groups.

	Type 2 Diabetes n = 16601	LADA n = 437	Type 1 Diabetes n = 34	P			
				Among groups	Type 2 diabetes vs LADA	LADA vs Type 1 diabetes	Type 2 diabetes vs Type 1 diabetes
**Female gender (%)**	55.8	51.6	48.5	0.151	0.088	0.869	0.500
**Family history of diabetes (%)**	68.3	71.9	87.9	**0.013**	0.115	0.065	**0.014**
**Age at diagnosis, mean ± SD (years)**	46.9 ± 9.6	45.1 ± 9.6	40.8 ± 9.6	**<0.001**	**<0.001**	**0.014**	**<0.001**
**BMI, mean ± SD (Kg/m^2^)**	31.4 ± 6.2	30.7 ± 6.2	27.7 ± 5.2	**<0.001**	**0.019**	**0.007**	**0.001**
**Waist Circumference, mean ± SD (cm)**	100.7 ± 12.5	99.5 ± 14.0	90.2 ± 12.9	**<0.001**	0.059	**<0.001**	**<0.001**
**HbA1c, mean ± SD (mmol/mol)**	69.4 ± 23.0 (8.5% ± 2.1%)	72.7 ± 23.0 (8.8% ± 2.1%)	86.9± 27.3 (10.1%±2.5%)	**<0.001**	**0.003**	**0.001**	**<0.001**
**Total Cholesterol, mean ± SD (mmol/L)**	4.8 ± 1.2	4.9 ± 1.2	5.0 ± 1.1	0.175	0.135	0.480	0.260
**Systolic BP, mean ± SD (mmHg)**	131.2 ± 20.7	127.6 ± 20.3	117.9 ± 16.9	**<0.001**	**<0.001**	**0.008**	**<0.001**
**Diastolic BP, mean ± SD (mmHg)**	72.9 ± 10.3	72.8 ± 10.1	69.1 ± 6.8	0.110	0.759	**0.007**	**0.003**

### LADA features by autoantibody

64.9% of LADA subjects were positive for GADA only, 31.0% for anti-IA2 only and 4.1% for both autoantibodies. A trend for age at diagnosis and waist circumference similar to the one described in type 2 diabetes, LADA and type 1 diabetes was also found among LADA subjects when subdividing by positivity for anti-IA2, GADA or for both antibodies (p = 0.013 for trend of age at diagnosis and p = 0.011 for trend of waist circumference) (Table 2 and S3 Table).

**Table 2:** Features of subjects with LADA by antibody positivity.

	Anti-IA2 only n = 135	GADA only n = 284	Both anti-IA2 and GADA n = 18	P among groups
**Female gender (%)**	53.3	50.5	55.6	0.817
**Age at diagnosis, mean ± SD (years)**	46.8 ± 9.2	44.5 ± 9.5	41.2 ± 10.2	**0.013**
**BMI, mean ± SD (Kg/m^2^)**	30.8 ± 5.9	30.7 ± 6.3	28.5± 5.5	0.314
**Waist Circumference, mean ± SD (cm)**	100.0 ± 12.3	99.8 ± 14.7	89.8 ± 13.1	**0.011**
**HbA1c, mean ± SD (mmol/mol, %)**	70.5 ± 23.0 (8.6% ± 2.1%)	73.8 ± 23.0 (8.9% ± 2.1%)	69.4 ± 19.7 (8.5% ± 1.8%)	0.361
**Total Cholesterol, mean ± SD (mmol/L)**	4.7 ± 1.2	5.0 ± 1.2	4.8 ± 1.3	**0.042**
**Systolic BP, mean ± SD (mmHg)**	128.3 ± 18.6	127.6 ± 20.6	124.4 ± 28.5	0.741
**Diastolic BP, mean ± SD (mmHg)**	72.8 ± 10.4	72.8 ± 9.9	70.5 ± 9.7	0.637

When comparing LADA subgroups by autoantibody with subjects affected by type 1 and type 2 diabetes we found that subjects simultaneously positive for both anti-IA2 and GADA did not differ from subjects with type 1 diabetes. On the contrary, subjects positive for GADA only and subjects positive for anti-IA2 only significantly differed from type 1 diabetes for higher systolic blood pressure (p = 0.012 and p = 0.004 for GADA only and anti-IA2 only vs type 1 diabetes, respectively), waist circumference (p<0.001 and p<0.001), BMI (p = 0.009 and p = 0.005), age at diagnosis (p = 0.05 and p<0.001) and for lower HbA1c (p = 0.002 and p<0.001).

**Fig 1:**
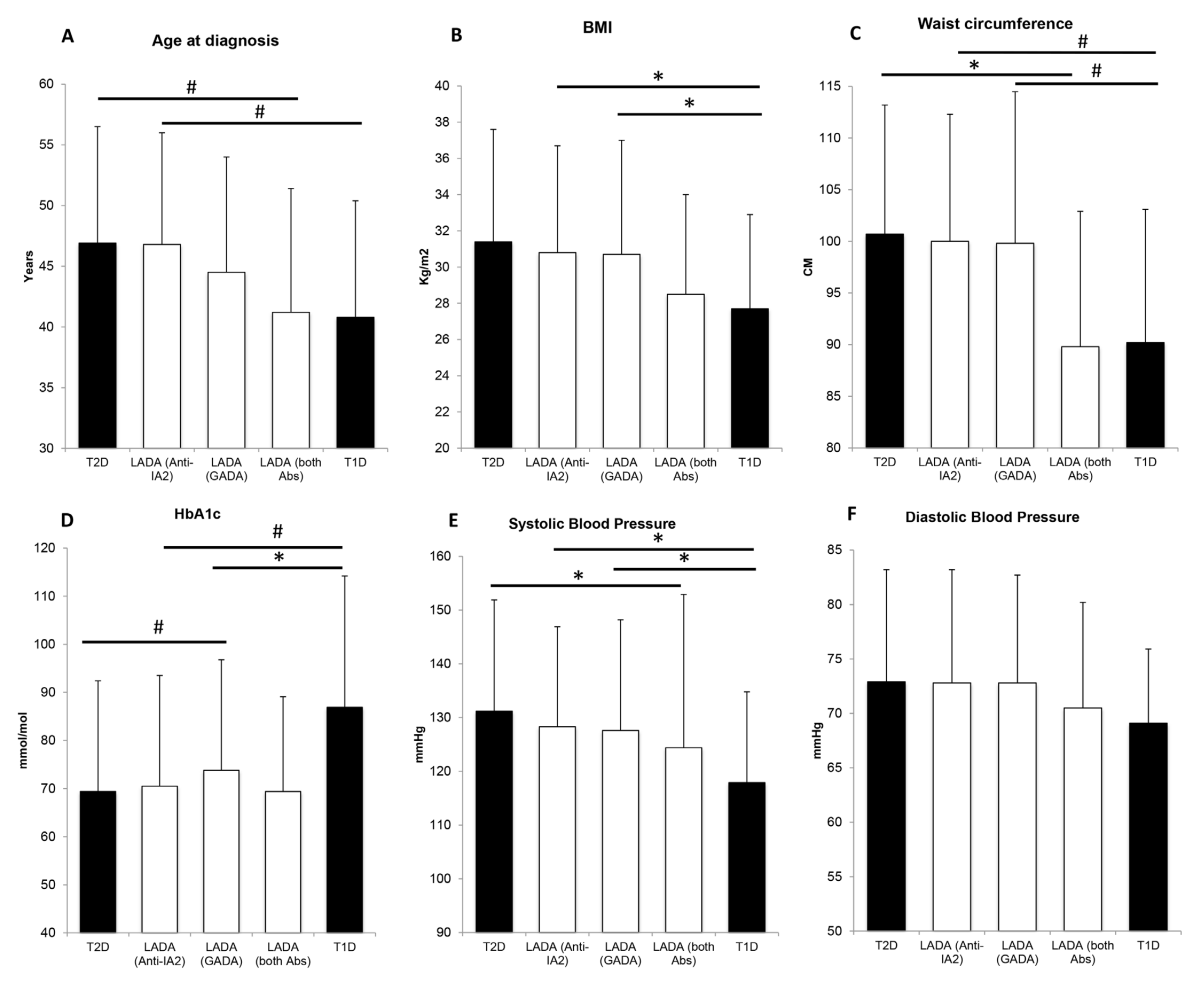
A-F Comparison between LADA subjects by antibody positivity (white columns) and type 1 and type 2 diabetes (black columns). Bars are for standard deviation. *p<0.05; #p<0.001. P-values for trend <0.001 for age at diagnosis, BMI, waist circumference, HbA1c and systolic blood pressure. T1D = type 1 diabetes; T2D = type 2 diabetes; LADA (Anti-IA2) = LADA subjects positive only for anti-IA2; LADA (GADA) = LADA subjects positive only for GADA; LADA (both Abs) = LADA subjects positive for both anti-IA2 and GADA.

Similarly, subjects positive only for anti-IA2 did not differ from patients with type 2 diabetes, while subjects positive for both autoantibodies did in lower systolic blood pressure (p = 0.008) and waist circumference (p = 0.004). Subjects positive only for GADA also differed from subjects with type 2 diabetes as they showed higher HbA1c (p<0.001), lower systolic blood pressure (p = 0.001) and age at diagnosis (p<0.001) (Fig 1A–1F).

### Factors influencing insulin therapy in subjects with LADA

At the time of data extraction 42.6% of LADA were on insulin. Glargine insulin was used by 63.2% of LADA subjects on insulin (detemir 8.2%); aspart insulin was used by 33.5% (lispro 11.0% glulisine 7.1%). 27.5% used premixed insulin. Before starting insulin therapy, metformin (alone or in combination) was used by 71.7% of LADA subjects, sulphonylureas by 44.2%, glinides by 1.2%, dipeptidyl peptidase-4 inhibitors by 48.0%, incretin analogues by 20.4% and acarbose by 1.9%.

To identify factors influencing time to insulin therapy in LADA we subdivided our population of LADA subjects into four groups according to the elapsed time to insulin therapy. 167 subjects were not included in this sub-analysis because they did not start insulin before data extraction and had a disease duration shorter than 10 years Among the remaining 270 subjects with LADA, 10 (3.7%) started insulin during the first year from diagnosis (<1y group), 49 (18.1%) between the first and the fifth year after diagnosis (1-5ys group), 60 (22.2%) between the fifth and the tenth year after diagnosis (5-10ys group) and 151 (55.9%) did not start insulin therapy within ten years from diagnosis (>10ys group).

**Fig 2:**
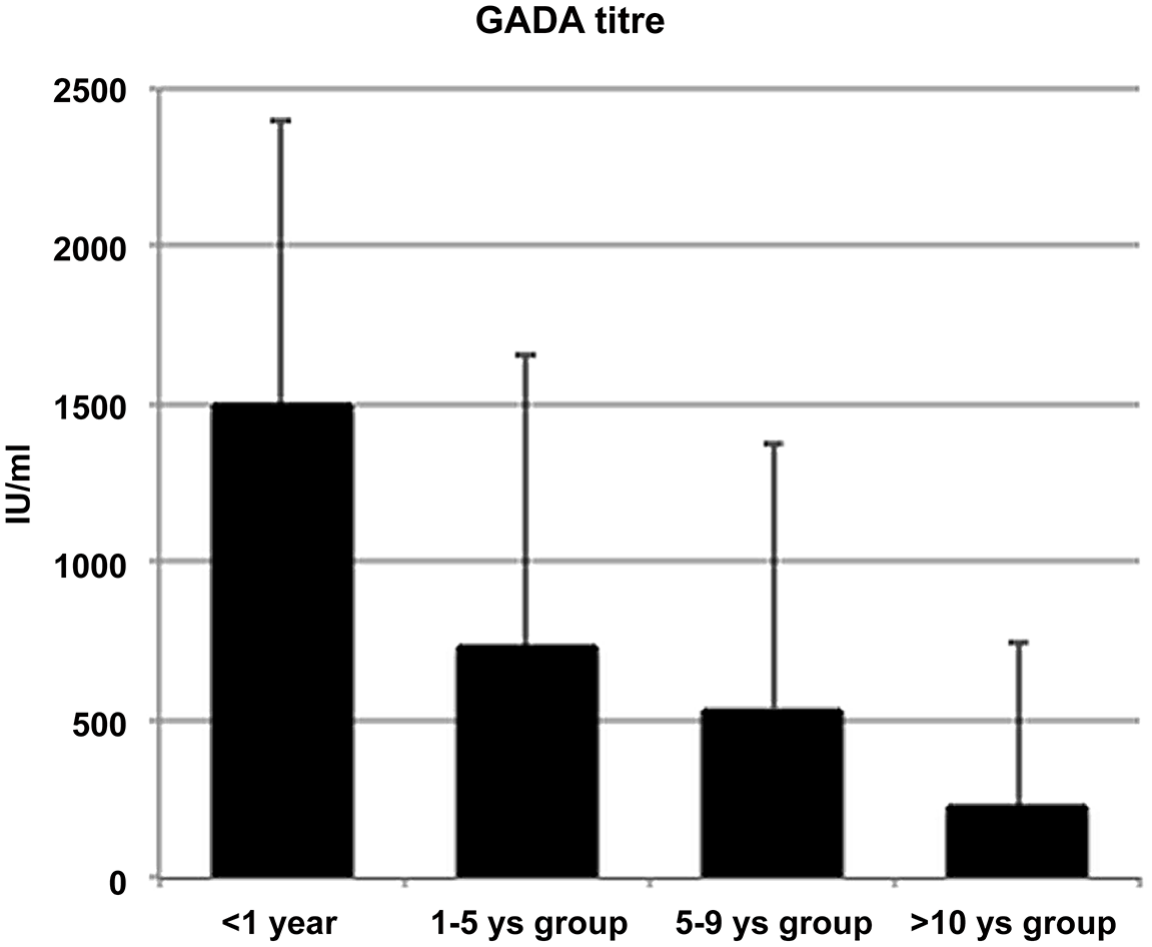
GADA titre in LADA subjects by groups of time to insulin. Bars are for standard deviation. p<0.001 for trend.

<1y group showed lower age at diagnosis (36.0 ± 6.5 years) than 1-5ys (43.42 ± 9.9 years, p = 0.008), 5-10ys (42.6 ± 8.4 years, p = 0.021) and >10ys groups (44.0 ± 9.0 years, p = 0.007); they also had lower waist circumference than 5-10ys (84.7 ± 11.2 cm vs 100.4 ± 13.6 cm, p = 0.021) and >10ys groups (101.3 ± 13.4, p<0.001) and lower BMI than >10ys group (26.9 ± 5.6 Kg/m^2^ vs 30.8 ± 5.6, p = 0.036). Mean HbA1c at diagnosis was significantly higher in 5-10ys group than in >10ys group (9.8% ± 2.1 vs9.0 ± 1,9, p = 0.004), but did not differs between the other groups. A significant trend in both autoantibodies titre was found: serum levels of GADA (Fig 2) and anti-IA2 significantly (p<0.001 for both) and gradually decreased from <1y group to >10ys group (p<0.001).

No other significant differences were found between LADA groups by time to insulin therapy.

## Discussion

Although there is ample data about the prevalence of diabetes in the Middle East, little is known about features of autoimmune diabetes in this region [1]. The knowledge of pathophysiology and characteristics of the disease is extremely important to implement nationwide preventive strategies and to correctly treat subjects affected by diabetes, allowing safe and effective therapies [16]. In particular, there is good evidence to show the importance of treating subjects affected by LADA differently from type 2 diabetes. Consistent data from randomized clinical trials showed the importance of an early initiation of insulin therapy in LADA avoiding the use of secretagogues like sulphonylureas [17]. To the best of our knowledge, this is the first study describing adult-onset autoimmune diabetes in the UAE, an affluent Gulf state with one of the highest comparative prevalence figures of diabetes worldwide and that will face an impressive increase in the number of diabetes diagnosis in the next few years.

As in Europe [11], in the UAE LADA appears to be the most prevalent form of adult-onset autoimmune diabetes. In the past, subjects affected by LADA have been often referred as phenotypic type 2 diabetes patients positive for at least one autoantibody [18], but some lines of evidence suggest a broad clinical phenotype in LADA [11,19,20]. Our data show that, as a form of autoimmune diabetes, clinical features of LADA are different from those of type 2 diabetes. In this large cohort LADA features were halfway between type 2 and type 1 diabetes for HbA1c at diagnosis, BMI, waist circumference and systolic blood pressure. These results are in agreement with previous data from European and Asian population [13,21] and also support the “*end of the rainbow*” hypothesis [22] suggesting that the different subtypes of adult-onset diabetes are not completely distinct diseases, but they form a continuum of varying severity of immune and metabolic dysfunction [23].

In this study we also looked at the possible influence of different patterns of antibody positivity on clinical features of subjects affected by LADA. Recently in a small cohort of LADA subjects the prevalence of metabolic syndrome components was related to the simultaneous positivity for different autoantibodies [24]. In our large cohort we found a higher prevalence of LADA subjects positive for anti-IA2 only than those described in Europe [11]. For the first time we showed that positivity for anti-IA2 only was associated with a clinical phenotype more similar to that found in type 2 diabetes, while the few subjects showing simultaneous positivity for GADA and anti-IA2 were more similar to type 1 diabetes. These data show that different pattern of autoantibody positivity can contribute to the clinical phenotype of subjects affected by LADA. Thus the measurement of autoantibodies in subjects suspected to have autoimmune diabetes has not only a diagnostic value, but can also identify subjects with different phenotypes. This should encourage studies describing new antibodies and new patterns of antibody positivity related to autoimmune diabetes.

Finally, we also looked at clinical features characterizing subjects with different time towards insulin requirement. We found that features differentiating subjects requiring an early initiation of insulin therapy were lower age at diagnosis, higher GADA titer, lower BMI and waist circumference. While we acknowledge that the low number of subjects that started insulin therapy within the first year from the diagnosis is a limit of our study, these results are in agreement with previous data showing an inverse relationship between GADA titers and C-peptide levels [25,26] and a higher incidence of insulin treatment in patients with higher GADA levels [27]. Moreover, more pronounced traits of insulin deficiency were described in patients with higher GADA titer [9]. On the contrary, before us Maioli et al. found that BMI, number of positive autoantibodies and HLA DRB1-DQB1 genotypes at high risk for type 1 diabetes were associated with the progression towards insulin requirement [28], but the association between GADA and insulin therapy was not significant after a follow-up period of four years as well as in the UKPDS 77 after a follow-up of 6 years [29]. Our results are derived from a dataset including subjects with a disease duration longer than 10 years, the longest one to date available in the literature for subjects with LADA.

There are some limitations to be considered when interpreting our results. The exclusion of one thousand twenty-nine subjects from the original dataset could represent a selection bias, but this is mostly due to the exclusion of subjects older than 70 years at the time of the diagnosis. Since we aimed to the characterization of autoimmune diabetes, which is usually diagnosed before 70 years of age [11], the study of subjects older than 70 years was beyond the scope of this paper. Furthermore, the number of subjects excluded represents a small proportion of the overall population. Residual beta-cell function could also influence the time to insulin therapy in subjects with LADA and we acknowledge that in this study we did not report data about c-peptide (a measure of beta-cell function), as values were available only for a small percentage of LADA subjects (11.9%). However, the available c-peptide data overall showed good residual function of beta cells in LADA subjects (mean values ± SD: 0.85 ± 0.72 nmol/L). Since no data are available about antihypertensive and lipid lowering agents, results about blood pressure and cholesterol levels should be interpreted with caution. We measured GADA and anti-IA2 serum levels, but we did not measure other autoantibodies that have been associated with adult-onset autoimmune diabetes as zinc-transporter 8 autoantibodies [30]. Further studies including a complete spectrum of diabetes-related antibodies will deeper investigate the influence of different patterns of autoantibody positivity on clinical features of subjects with autoimmune diabetes.

In conclusion, to the best of our knowledge, this the first study describing presence and features of adult-onset autoimmune diabetes in the UAE. Our study confirms that in this population LADA subjects are clinically different from type 2 and type 1 diabetes and suggests that the clinical phenotype can depend on different patterns of antibody positivity influencing the time to insulin requirement.

## Supporting Information

S1 File (XLSX)

S1 Table Hometowns of patients registered at the ICLDC. (DOCX)

S2 Table Antibody titre by type of autoimmune diabetes and by gender. GADA = autoantibodies to Glutamic Acid Decarboxylase; Anti-IA2 = autoantibodies to Islet Antigen 2. Unit of measure is IU/ml for both antibodies. *p = 0.001 vs LADA; ^#^p<0.001 vs LADA; ^p = 0.007 vs LADA; °p = 0.004.
(DOCX)

S3 Table Between groups p-values by antibody positivity. (DOCX)
