# Correction: Latent Autoimmune Diabetes in Adults in the United Arab Emirates: Clinical Features and Factors Related to Insulin-Requirement

**DOI:** 10.1371/journal.pone.0137152

**Published:** 2015-08-27

**Authors:** The PLOS ONE Staff

## Notice of Republication

This article was republished on August 14, 2015, to remove confidential or copyrighted material. Please download this article again to view the correct version.
