# Supplementary material for: Latent Autoimmune Diabetes in Adults in the United Arab Emirates: Clinical Features and Factors Related to Insulin-Requirement
Source: PLoS One. 2015 Aug 7;10(8):e0131837. doi: 10.1371/journal.pone.0131837 (PMC4529198; doi:10.1371/journal.pone.0131837)
Supplement: S1 Table — (DOCX) [file pone.0131837.s002.docx]

| **Location** | **% of patients registered** |
| --- | --- |
| Abu Dhabi | 52.0% |
| Al Ain | 26.0% |
| International | 5.6% |
| Bani Yas | 3.2% |
| Ras Al Khaimah | 2.9% |
| Sharjah | 2.1% |
| Al Shamkha | 2.1% |
| Dubai | 2.1% |
| Fujairah | 1.9% |
| Western Region | 1.4% |
| Ajman | 0.4% |
| Um Al Quwain | 0.3% |
| **Grand Total** | **100.00%** |

**S1 table. Hometowns of patients registered at the ICLDC**
