# Supplementary material for: Latent Autoimmune Diabetes in Adults in the United Arab Emirates: Clinical Features and Factors Related to Insulin-Requirement
Source: PLoS One. 2015 Aug 7;10(8):e0131837. doi: 10.1371/journal.pone.0131837 (PMC4529198; doi:10.1371/journal.pone.0131837)
Supplement: S2 Table — GADA = autoantibodies to Glutamic Acid Decarboxylase; Anti-IA2 = autoantibodies to Islet Antigen 2. Unit of measure is IU/ml for both antibodies. *p = 0.001 vs LADA; #p<0.001 vs LADA; ^p = 0.007 vs LADA; °p = 0.004. (DOCX) [file pone.0131837.s003.docx]

**S2 table.** **Antibody titre by type of autoimmune diabetes and by gender**. GADA= autoantibodies to Glutamic Acid Decarboxylase; Anti-IA2= autoantibodies to Islet Antigen 2. Unit of measure is IU/ml for both antibodies.

*p=0.001 vs LADA; ^#^p<0.001 vs LADA; ^p=0.007 vs LADA; °p=0.004.

|  | Overall | Males | Females |
| --- | --- | --- | --- |
| Type 1 Diabetes   - GADA - Anti-IA2 | 785.2 ± 918.9*  378.6 ± 997.8^#^ | 905.8 ± 953.0°  327.8 ± 1015.6^ | 657.1 ± 893.6  432.5 ± 1008.7^#^ |
| LADA   - GADA - Anti-IA2 | 361.1 ± 707.4  48.3 ± 297.7 | 367.2 ± 706.7  51.0 ± 314.6 | 355.4 ± 709.6  45.8 ± 281.6 |
