# Supplementary material for: Latent Autoimmune Diabetes in Adults in the United Arab Emirates: Clinical Features and Factors Related to Insulin-Requirement
Source: PLoS One. 2015 Aug 7;10(8):e0131837. doi: 10.1371/journal.pone.0131837 (PMC4529198; doi:10.1371/journal.pone.0131837)
Supplement: S3 Table — (DOCX) [file pone.0131837.s004.docx]

|  | **P** | | |
| --- | --- | --- | --- |
|  | Anti-IA2 vs GADA | Anti-IA2 vs both | GADA vs both |
| **Female gender** | 0.568 | 0.859 | 0.669 |
| **Age at diagnosis** | **0.005** | 0.101 | 0.423 |
| **BMI** | 0.817 | 0.155 | 0.221 |
| **Waist Circumference** | 0.634 | **0.033** | 0.051 |
| **HbA1c** | 0.145 | 0.477 | 0.190 |
| **Total Cholesterol** | **0.010** | 0.741 | 0.626 |
| **Systolic BP** | 0.391 | **0.018** | **0.029** |
| **Diastolic BP** | 0.817 | 0.367 | 0.298 |

**S3 table. Between groups p-values by antibody positivity**
